# Minimally invasive approach to peritonitis from left colonic perforation: a retrospective multicenter observational study

**DOI:** 10.1007/s00464-025-11611-w

**Published:** 2025-02-18

**Authors:** Marco Ceresoli, Carola Anna Paolina Ferro, Antonio La Greca, Stefano Piero Bernardo Cioffi, Alan Biloslavo, Mauro Podda, Federico Coccolini, the Study Collaborative Group, Lorena Massante, Dario Palmisano, Marco Braga, Valerio Cozza, Valeria Fico, Giuseppe Tropeano, Gaia Altieri, Riccardo Damiani, Michele Altomare, Francesco Virdis, Stefania Cimbanassi, Paola Germani, Adriana Gioia, Davide Ciriotto, Valentina Murzi, Tiziana Pilia, Alessia Dessì, Adolfo Pisanu, Rossella Facchin, Giuseppe Zocco, Camilla Cremonini, Dario Tartaglia

**Affiliations:** 1 School of Medicine and Surgery, University of Milano-Bicocca, Monza, Italy; 2 Department of General and Emergency Surgery, Fondazione IRCCS San Gerardo Dei Tintori, Monza, Italy; 3 Emergency Surgery and Trauma Unit, Fondazione IRCSS Policlinico Universitario A Gemelli Roma, Rome, Italy; 4 Chirurgia Generale - Trauma Team ASST Grande Ospedale Metropolitano Niguarda, Milan, Italy; 5 Department of General Surgery, Cattinara University Hospital, Trieste, Italy; 6 Department of Surgical Science, University of Cagliari, Cagliari, Italy; 7 General, Emergency and Trauma Surgery Department, Pisa University Hospital, Pisa, Italy

**Keywords:** Laparoscopy, Emergency surgery, Colic perforation, Minimally invasive surgery

## Abstract

**Background:**

Laparoscopic procedures have nowadays become the gold standard in multiple abdominal diseases, but in the emergency setting, especially in major surgery, laparoscopy still represents an obstacle for most surgeons. This multicentric study aims to define the surgical approach for emergency resective surgery in left colonic perforation peritonitis, determine the factors influencing the choice between MIS and open surgery, and identify factors associated with laparotomic conversion.

**Methods:**

Retrospective data from 516 patients treated for left colonic perforation between January 2019 and December 2023 across six Italian centers was collected. Variables analyzed included patient demographics, disease and surgical characteristics, post-operative complications, and long-term outcomes. Univariate and multivariate regression models were performed to identify factors associated with surgical choice and likelihood of conversion.

**Results:**

Only 24.5% of patients underwent laparoscopic approach, with a conversion rate of 52.8%. MIS was associated to lower CCI and MPI and less severe septic status at arrival. MIS was mostly performed by colorectal surgeons, compared to acute care surgeons. The post-operative outcome, considering LOS, ICU admission, post-operative complications and mortality rate, was better in the MIS group. The multivariate regression model revealed that CCI, MPI, and night-time surgery were negatively associated with MIS while colorectal surgeons had a positive association. Laparotomic conversion more commonly occurred in older patients with a higher MPI and CCI and more severe septic status; these patients had a worse post-operative outcome. MPI was the only factor with statistical significance in the univariate analysis, associated with conversion.

**Conclusion:**

Although MIS is associated with better post-operative outcomes, it is underutilized in the emergency setting. Patients requiring laparotomic conversion had higher morbidity and mortality compared to those who underwent successful laparoscopy. Limiting factors for MIS include logistic factors, patient severity, and surgical skills, therefore careful patient selection and surgical training is crucial.

Left colonic perforation remains a critical surgical emergency, often associated with significant morbidity and mortality, which requires an urgent surgical intervention due to the possible rapid deterioration of patients from peritonitis to septic shock. Traditionally, open surgery has been the mainstay of treatment for this condition, however with the spread of minimally invasive surgical techniques, such as laparoscopy a debate about which is the best surgical approach has been opened.

Laparoscopy has been widely recognized for its benefits in terms of morbidity and mortality, including shorter length of stay and reduced post-operative pain, indeed it has become the gold standard in multiple elective surgeries [1–3]. Indeed, nowadays elective surgery is characterized by a wide use of minimally invasive surgery (MIS), including robotics, for major elective abdominal procedures, especially in colorectal disease, such as diverticulitis [4–6]. However, in the emergency setting, especially in case of major surgery, laparoscopy still represents an underused technique for most patients [4]. This is often due to the complexity of the patients’ clinical conditions, including the severity of abdominal and systemic conditions and patient instability, and the possible limited experience of surgeons in laparoscopy [4]. In the emergency setting MIS is primarily used for minor surgeries, such as appendicectomy or cholecystectomy [7–9]. In fact, most of the guidelines for major abdominal emergencies only consider laparoscopy in centers where there is sufficient experience, where technical skills and equipment are available, but are not routinely suggested [10]. On the other hand, current literature indicates that MIS offers these advantages also in the emergency setting [2, 6]. Indeed, literature suggests that there is a reported lower use of MIS for major abdominal surgeries [11], although the benefits of laparoscopic surgery, such as lower bleeding, lower LOS and lower morbidity and mortality, are maintained in the emergency setting, also in high-risk patients [7, 12, 13].

In the case of left colonic perforations, laparoscopy permits a thorough exploration of the abdominal cavity to identify the exact site of perforation and allows to perform the same type of surgery as an open approach does. Nowadays most guidelines recommend MIS approach in colonic perforation, but only in cases where the operator’s skills and the patient’s conditions are deemed adequate [10, 14, 15].

The aim of our study was to define benefits and disadvantages of the different surgical approaches in peritonitis from left colonic perforation and to identify the variables that influence the decision to initiate with MIS, the likelihood of converting from laparoscopic to open surgery, and the outcomes associated with each approach.

## Materials and methods

This is a retrospective multicenter study including six different high volume emergency general surgery centers in Italy. All the participating centers are tertiary level academic high volume teaching hospitals, with dedicated acute care services and dedicated colorectal surgical units (each hospital performed more than 100 elective colorectal resections per year and more than 1000 emergency surgery procedures per year). All patients with a diagnosis of left colonic perforation or abscess and who underwent emergency resection between the 1st of January 2019 and the 31st of December 2023 were retrieved. We excluded patients who were diagnosed with peritonitis from a right colonic perforation, patients < 18 years old, those who didn’t undergo resective surgery, those with traumatic perforation or perforation as a complication of an elective surgery, and patients that were lost at follow up; iatrogenic perforations after endoscopic procedures were included.

The population was then divided into two categories, those who started surgery with a MIS approach (in this study we only considered laparoscopy, not robotic surgeries) and those who had laparotomic surgical approach. In the MIS population, we also considered the patients that completed a successful laparoscopic surgery separately from those that had to undergo laparoscopic to open conversion.

Left colonic perforation was defined as perforation with/or without abscess found anywhere from the distal transverse colon to the rectum, included. The diagnosis was based on pre-operative CT scan.

Indications for surgery included presence of peritonitis or of septic status, including presence of hemodynamic instability, or peritoneal abscesses that failed initially non operative management according to guidelines [15].

The analyzed variables were divided into: patient characteristics, such as Clinical Frailty Scale (CFS) and Charlson Comorbidity Index (CCI); disease characteristics, such as septic status and Mannheim Peritonitis Index (MPI). Surgery characteristics (timing of surgery, type and age of the surgeon; surgical technique adopted); operating surgeons were classified as colorectal (> 50% of working time dedicated to elective colorectal surgery), acute care (> 50% of working time dedicated to emergency general surgery and trauma) or other; moreover surgeons were defined as senior if > 40 years old and if they performed > 50 resective colorectal surgeries/year (only for colorectal). Post-operative complications were classified using the Clavien–Dindo classification (CD > IIIa were recorded as severe complications); and long-term outcomes one year after primary surgery including rate of readmissions, of reinterventions, and mortality rate.

Qualitative data were summarized using frequencies and percentages, while quantitative data were summed up with median and interquartile range. A univariate and a multivariate regression model were created for the initial MIS approach and to research the factors associated with successful laparoscopy vs converted cases. Multiple regression models were created considering variables significantly associated with the outcome of interest at the univariate analysis. Statistical significance was considered as p value ≤ 0.05. Data was analyzed using SPSS Statistics (IBM Corp. Released 2023. IBM SPSS Statistics for Windows, Version 29.0.2.0 Armonk, NY: IBM Corp).

## Results

### Descriptive statistics of the whole population

A total of 516 patients were analyzed over a 5-year time span in the 6 selected centers. The pre-operative and operative variables are represented in Table 1.

**Table 1:** Pre-operative and operative characteristics of the whole population

		Open		Laparoscopy		P	Total	
		N/median	%/IQR	N/median	%/IQR		N/median	%/IQR
Sex	F	217	55.8%	62	48.8%	0.172	279	54.1%
	M	172	44.2%	65	51.2%		237	45.9%
Age		72	60–81	64	53–74	** < 0.001**	70	58–79
BMI		24.98	22.62–28.30	24.69	23.04–27.60	0.756	24.82	22.79–28.00
Clinical frailty scale		4	3–5	3	2–4	** < 0.001**	3	3–5
Charlson comorbidity index		3	1–5	1	0–3	** < 0.001**	3	1–5
Diagnosis	Cancer perforation	34	8.7%	6	4.7%	** < 0.001**	40	7.8%
	Diverticular abscess	58	14.9%	47	37.0%		105	20.3%
	Diverticular perforation	254	65.3%	66	52.0%		320	62.0%
	Iatrogenic perforation *	5	1.3%	4	3.1%		9	1.7%
	Ischemic perforation	27	6.9%	1	0.8%		28	5.4%
	Other **	11	2.8%	3	2.4%		14	2.7%
Mannheim peritonitis index		22	16–28	15	10–20	** < 0.001**	20	15–26
Septic status	No	220	56.6%	106	83.5%	** < 0.001**	326	63.2%
	Septic Shock	67	17.2%	5	3.9%		72	14.0%
	Severe Sepsis	102	26.2%	16	12.6%		118	22.9%
Diagnosis	Peritoneal Abscess	58	14.9%	47	37.0%	** < 0.001**	105	20.3%
	Peritonitis	331	85.1%	80	63.0%		411	79.7%
Intervention performed	Open abdomen + end stoma	44	11.3%	1	0.8%	** < 0.001**	45	8.7%
	Open abdomen + primary anastomosis	9	2.3%	3	2.4%		12	2.3%
	Resection + end stoma	224	57.6%	25	19.7%		249	48.3%
	Resection + primary anastomosis	112	28.8%	98	77.2%		210	40.7%
Time of surgery	Afternoon	157	40.4%	48	37.8%	** < 0.001**	205	39.7%
	Morning	81	20.8%	62	48.8%		143	27.7%
	Night	151	38.8%	17	13.4%		168	32.6%
Period of surgery	Holiday	10	2.6%	2	1.6%	0.616	12	2.3%
	Week	279	71.7%	94	74.0%		373	72.3%
	Weekend	100	25.7%	31	24.4%		131	25.4%
Operating surgeon	Acute Care	218	76.0%	69	24.0%	**0.045**	287	55.6%
	Colorectal	102	68.0%	48	32.0%		150	29.1%
	Other	69	87.3%	10	12.7%		79	15.3%
Operating surgeon	< 40 years old	136	75.6%	44	24.4%	0.948	180	34.9%
	≥ 40 years old	253	75.3%	83	24.7%		336	65.1%
Operating time (minutes)		175	130–225	255	190–300	**0.04**	190	140–255
Protective stoma		49	12.6%	28	22.0%	**0.023**	77	14.9%

Bold values are statistically significant (p < 0.05)

*Includes patients with perforation following endoscopic procedures

**Includes for example perforation due to foreign objects, decubitus of fecalomas, etc.

279 (54.1%) were females with a median age of 70 years old (IQR 58–79). The median CFS was 3 (IQR 3–5) and CCI was 3 (IQR 1–5). Most of the patients were affected by diverticular perforation (62%, *n* = 320), followed by diverticular abscess (20.3%, *n* = 105). The median MPI was of 20 (IQR 15–26).

Most patients underwent an open approach (75.4%, *n* = 389) compared to laparoscopic approach (24.5%, *n *= 127), of which 52.8% (*n* = 67) required conversion to open surgery. Most patients underwent Hartmann’s procedure (57.0%, *n* = 294), followed by resection and primary anastomosis (43.0%, *n* = 222); including patients with open abdomen and primary closure. Surgery was more often performed in the afternoon (39.7%, *n* = 205) and in weekdays (72.3%, *n* = 373).

Table 2 summarizes the post-operative characteristics of the patients.

**Table 2:** Post-operative characteristics of the whole population

		Open		Laparoscopy		p	Total	
		N/median	%/IQR	N/median	%/IQR		N/median	%/IQR
ICU admission		178	46.0%	20	15.7%	** < 0.001**	198	38.5%
ICU days		4	2–10	1	1–5	**0.002**	4	2–9
Ventilation days		2	1–6	1	0–2	**0.033**	2	1–6
LOS (days)		13	8–24	14	9–20	0.877	13	8–22
Stoma at discharge	No	62	15.9%	67	52.8%	** < 0.001**	129	25.0%
	End stoma	278	71.5%	33	26.0%		311	60.3%
	Protective stoma	49	12.6%	27	21.3%		76	14.7%
Reintervention		66	17.0%	21	16.5%	0.910	87	16.9%
Complications		273	70.2%	66	52.0%	** < 0.001**	339	65.7%
Clavien–Dindo Grade	0	116	29.8%	61	48.0%	** < 0.001**	177	34.3%
	I	42	10.8%	24	18.9%		66	12.8%
	II	100	25.7%	19	15.0%		119	23.1%
	IIIa	10	2.6%	4	3.1%		14	2.7%
	IIIb	16	4.1%	11	8.7%		27	5.2%
	IVa	19	4.9%	4	3.1%		23	4.5%
	IVb	9	2.3%	0	0.0%		9	1.7%
	V	77	19.8%	4	3.1%		81	15.7%
Death		77	19.8%	4	3.1%	** < 0.001**	81	15.7%
SSI within 30 days		53	13.6%	12	9.4%	0.181	65	12.6%
Abdominal abscess		41	10.5%	9	7.1%	0.253	50	9.7%
Anastomotic leak		16	4.1%	12	9.4%	**0.021**	28	5.4%
Respiratory infections		59	15.2%	9	7.1%	**0.019**	68	13.2%
Renal failure		48	12.3%	1	0.8%	** < 0.001**	49	9.5%
Cardiovascular complications		58	14.9%	5	3.9%	** < 0.001**	63	12.2%
Urinary infections		22	5.7%	1	0.8%	** < 0.021**	23	4.5%
Readmission		56	18.7%	17	14.5%	0.311	73	17.5%

Bold values are statistically significant (p < 0.05)

The median LOS was of 14 days (IQR 8–22), 38.5% (*n* = 198) were admitted in ICU with a median ICU-LOS of 4 (IQR 2–8) days. The 30-day mortality rate was 15.7% (*n* = 81). Post-operative complications were recorded in 65.7% of patients (*n* = 339) with 29.8% of severe complications (*n* = 154) and 17.2% reinterventions (*n* = 89). Sensitivity analyses were also performed to evaluate the heterogeneity among centers, and they did not show any significant difference.

### Laparoscopic vs open approach: descriptive statistics

MIS patients were more often non-septic (83.5%, *n* = 103) and presented with fewer cases of peritonitis (63%, *n* = 80) while the open surgery group was characterized by a higher MPI (22, IQR 16–28). Among patients approached with MIS resection with primary anastomosis was the most common intervention (*n* = 98, 77.2%) while in the open group 57.6% of patients underwent Hartmann’s procedure (*n* = 224).

The overall LOS was similar between the two groups while the total rate of complications was higher in the open surgery group (70.2%, *n* = 273 vs 52%, *n* = 66). The only complication that appeared more frequently in the laparoscopic group was anastomotic leak (9.4%, *n* = 12 vs 4.1%, *n* = 16).

A summary of the characteristics of each population is represented in Tables 1 and 2.

Multiple regression analysis identified the key factors associated to MIS adoption (summarized in Table 3). Higher CCI (OR = 0.797; 95% CI 0.661–0.960), higher MPI (OR = 0.956; 95% CI 0.919–0.996), and night-time surgery (OR = 0.213; 95% CI 0.095–0.480) were negatively associated with MIS approach; surgery performed by a colorectal surgeon was positively associated with MIS (OR = 3.047; 95% CI 1.297–7.157).

**Table 3:** Univariate and multivariate analysis of factors associated to the use of laparoscopic approach vs open surgery

		Univariate analysis				Multivariate analysis			
		OR	95% CI		p-value	OR	95% CI		p-value
Sex		0.756	0.506	1.129	0.172				
Age		0.967	0.953	0.980	**< 0.001**	1.008	0.981	1.035	0.576
BMI		0.993	0.948	1.040	0.765				
Clinical Frailty Scale		0.687	0.586	0.804	**< 0.001**	0.898	0.716	1.127	0.354
Charlson Comorbidity index		0.778	0.709	0.853	**< 0.001**	0.797	0.661	0.960	**0.017**
Septic status	No severe sepsis				**< 0.001**				0.137
	Severe sepsis	0.326	0.183	0.579	**< 0.001**	0.653	0.320	1.330	0.240
	Septic shock	0.155	0.061	0.396	**< 0.001**	0.346	0.113	1.060	0.063
Diagnosis	Peritonitis	0.298	0.189	0.470	**< 0.001**	0.637	0.335	1.210	0.168
	Abscess	3.353	2.126	5.288	**< 0.001**				
Weekend or holiday		1.123	0.713	1.768	0.616				
Time of the day	Morning								
	Afternoon	0.399	0.252	0.634	**< 0.001**	0.646	0.350	1.193	0.162
	Night	0.147	0.081	0.268	**< 0.001**	0.213	0.095	0.480	**< 0.001**
Type of surgeon	Acute care								
	Colorectal	1.487	1.060	2.302	**0.045**	3.047	1.297	7.157	**0.011**
	Other	0.458	0.224	0.937	**0.033**	0.696	0.303	1.598	0.392
Operating surgeon > 40 y.o		1.014	0.666	1.544	0.948				
Mannheim Peritonitis Index		0.908	0.882	0.934	**< 0.001**	0.956	0.917	0.996	**0.033**

Bold values are statistically significant (p < 0.05)

### Successful vs converted laparoscopy: descriptive statistics

Tables 4 and 5 describe characteristics of patients underwent successful laparoscopy compared with patients converted to open surgery.

**Table 4:** Conversion vs successful MIS pre-operative and operative characteristics

		Conversion		Successful MIS		P
		N/median	%/IQR	N/median	%/IQR	
Sex	F	32	47.8%	30	50.0%	0.801
	M	35	52.2%	30	50.0%	
Age		65	56–75	61	52–73	0.072
BMI		24.69	22.89 −27.76	24.69	23.15- 27.34	0.875
Clinical frailty scale		3	2–3	3	2–4	0.818
Charlson comorbidity index		2	1–3	1	0–3	0.220
Diagnosis	Cancer perforation	5	7.5%	1	1.7%	0.429
	Diverticular abscess	23	34.3%	24	40.0%	
	Diverticular perforation	35	52.2%	31	51.7%	
	Iatrogenic perforation *	1	1.5%	3	5.0%	
	Ischemic perforation	1	1.5%	0	0.0%	
	Other **	2	3.0%	1	1.7%	
Mannheim peritonitis index		16	11–23	15	10–16	**0.010**
Septic status	No	52	77.6%	54	90.0%	0.059
	Septic shock	5	7.5%	0	0.0%	
	Severe sepsis	10	14.9%	6	10.0%	
Diagnosis	Peritoneal abscess	23	34.3%	24	40.0%	0.286
	Peritonitis	44	65.7%	36	60.0%	
Intervention performed	Open abdomen + end stoma	1	1.5%	0	0.0%	0.065
	Open abdomen + primary anastomosis	3	4.5%	0	0.0%	
	Resection + end stoma	17	25.4%	8	13.3%	
	Resection + primary anastomosis	46	68.7%	52	86.7%	
Time of surgery	Morning	30	44.8%	32	53.3%	0.108
	Afternoon	24	35.8%	24	40.0%	
	Night	13	19.4%	4	6.7%	
Period of surgery	Holiday	2	3.0%	0	0.0%	0.361
	Week	50	74.6%	44	73.3%	
	Weekend	15	22.4%	16	26.7%	
Operating surgeon	Acute Care	35	52.2%	34	56.7%	0.191
	Colorectal	24	35.8%	24	40.0%	
	Other	8	11.9%	2	3.3%	

Bold value indicates statistical significance (p < 0.05)

*Refers to patients with perforation following endoscopic procedures

**Includes for example perforation due to foreign objects, stercoral perforation, etc.

**Table 5:** Conversion vs successful MIS patient’s post-operative characteristics

		Conversion		Successful MIS		P
		N/median	%/IQR	N/median	%/IQR	
ICU admission		16	23.9%	4	6.7%	**0.008**
ICU days		1	1–5	2	1–6	0.914
N. of ventilation days		1	1–3	0	0–0	0.207
LOS (days)		16	12–21	11	8–18	**0.002**
Stoma at discharge	No	30	44.8%	37	61.7%	0.276
	End stoma	22	32.8%	11	18.3%	
	Protective stoma	15	22.4%	12	20.0%	
Reintervention		13	19.4%	9	15.0%	0.659
Complications		40	59.7%	26	43.3%	0.065
Clavien–Dindo Grade	I	11	16.4%	13	21.7%	0.173
	II	13	19.4%	6	10.0%	
	IIIa	3	4.5%	1	1.7%	
	IIIb	7	10.4%	4	6.7%	
	IVa	2	3.0%	2	3.3%	
	V	4	6.0%	0	0.0%	
Death		4	6.0%	0	0.0%	0.054
SSI within 30 days		11	16.4%	1	1.7%	**0.005**
Abdominal abscess		7	10.4%	2	3.3%	0.119
Anastomotic leak		8	11.9%	4	6.7%	0.310
Respiratory infections		6	9.0%	3	5.0%	0.386
Renal failure		1	1.5%	0	0.0%	0.342
Cardiovascular complications		3	4.5%	2	3.3%	0.742
Urinary infections		1	1.5%	0	0.0%	0.342
Readmission		9	14.8%	8	14.3%	0.875

Bold values are statistically significant (p < 0.05)

Converted patients were older (65 years old, IQR 58–75), with more comorbidities (CCI 2 IQR 1–3), and a higher MPI (16, IQR 11–23) compared to patients who completed laparoscopy successfully. Successful laparoscopy patients presented more often with a non-septic status (90%, *n* = 54).

Successful laparoscopy was more often associated to resection and primary anastomosis (86.7%, *n* = 52) than conversion (68.7%, *n* = 46).

23.9% of converted patients were admitted to the ICU (*n* = 16) compared to 6.7% of successful laparoscopy patients (*n* = 4). The overall LOS was of 16 days in the former group (IQR 12–21), longer than those of successful MIS (11 days, IQR 8–18). Converted patients were characterized by a greater number of complications (59.7%, *n* = 40), and of higher grade. Zero patients died in the successful MIS group compared to a 6% mortality rate (*n* = 4) in the converted group.

At the regression analysis the only factor associated with conversion to open surgery was the MPI (OR = 0.919, *p* = 0.001).

### Converted laparoscopy vs open surgery: descriptive statistics

Converted patients presented with a lower CCI (2, IQR 1–3) and MPI (16, IQR 11–23) compared to open surgery patients (respectively 3, IQR 1–5; 22, IQR 16–28). They more commonly underwent resection and primary anastomosis (68.7%, *n* = 46) while open surgery patients were subjected to Hartmann’s procedure most of the time (57.6%, *n* = 224).

In the post-operative period, open surgery patients were characterized by a higher degree of complications (70.2%, *n* = 273), LOS (14 days, IQR 9–20), and mortality rate (19.8%, *n* = 77) compared to converted patients (respectively 59.7%, *n* = 40; 16 days, IQR 12–21; 6%, *n* = 4).

## Discussion

The present study shows that MIS approach to left side colonic perforations and abscesses is adopted in a minority of cases. Patients treated with MIS suffer of a less severe disease and have better post-operative outcomes, with a reduced ICU stay, reduced complications and reduced mortality. Higher MPI, higher CCI, and night-time surgeries were negatively related to a MIS approach, colorectal operating surgeons were positively related.

When overlooking our results, what we can notice is the scarce use of laparoscopy in the emergency setting. Indeed only 24.5% of our patients started with a MIS approach, irrespectively of whether it was successful or not. As in the literature, also in the case of our study, patients who performed and completed the surgery in laparoscopy were characterized by a lower rate of complications, lower stay in the ICU, and lower mortality. This is both due to less invasive approach of laparoscopy, but also probably to the selection of patients that undergo MIS, which often present with a less severe septic status and hemodynamic instability.

In our cohort of patients, the only complication that more commonly occurred in the laparoscopic group was anastomotic leak. This can be explained by the fact that primary anastomoses completed in the open group were performed in a smaller number of patients, suggesting a higher selection of patients. Moreover, colorectal surgeons could be more prone to perform primary anastomoses due to their more complete experience in this type of procedures. Nonetheless we found that the number of reinterventions was equivalent in the two groups, so patients that underwent primary anastomosis did not undergo more reinterventions than those that performed Hartmann’s procedure. Moreover the number of overall complications and mortality were lower in this group. This implies that in primary anastomosis patients have better overall outcomes than patients with end stoma, despite the higher rate of anastomotic leak. Further studies to eliminate this selection bias and explore more in detail this aspect should be performed to obtain a more clear and truthful result about the rate of anastomotic leakage.

Patients that started with laparoscopy but had to undergo conversion to open surgery, had a higher morbidity and mortality compared to successful laparoscopy, but not worse than those who were subject to open surgery directly, in line with the current literature [16–18]. This could be implemented in the daily surgical choice as a sign that starting with laparoscopy should always be performed, except in cases of absolute contraindications, as converted patients still have a better outcome than open surgery patients.

Colorectal surgeons were more prone to perform MIS compared to acute care surgeons, which is supported by current literature [8, 13]. A recent survey on the use of MIS in emergency surgery all over the world reported that the main factors associated to laparoscopy were longer surgical experience and personal involvement in elective laparoscopic surgery [4]. Our results confirmed this finding, since nowadays nearly all elective colorectal resections are performed with laparoscopy [1, 2].

These data contribute to the ongoing debate about the organizational model of emergency surgery services and the need for a dedicated training pathway for acute care surgeons, which includes a specific focus on minimally invasive surgery. Moreover, these data suggest the need for a 'hybrid' model that allows emergency surgeons to be exposed also to elective cases in order to develop and enhance their skills in minimally invasive surgical techniques (both laparoscopy and robotic). We must acknowledge that our data refers to the Italian context where the type of surgical training is the same for everyone. However, in tertiary centers, which are the ones who participated to this study, there are different teams dedicated to surgical subspecialties. In the future it would be interesting to verify our results in a larger multicentric study involving also other countries in order to evaluate how different hospital organizations and different surgical training models actually impact on the rate of laparoscopy in emergency surgery and its outcomes.

The multiple regression analysis showed Charlson Comorbidity Index, Mannheim Peritonitis Index, night-time surgery as variables negatively correlated to MIS approach. Patients’ pre-operative conditions intuitively seem to be a determinant factor in the choice of surgical approach, indeed patients with a higher CCI, who are more likely to be older and have a greater amount of comorbidities, and a higher severity of the latter. Moreover patients in worse condition with worse peritonitis (higher MPI) were less likely to be operated using MIS. This result could be explained by a higher instability of the patients, being a relative contraindication to laparoscopy, especially if associated with a weaker experience of the surgeon in MIS. Also logistic factors had an influence on the choice of approach: patients operated during the night-time were more often subjected to open surgery. The reason for this is probably the scarcity of resources during the night compared to the daytime, when more assets are available in case of difficulty. Lastly, colorectal surgeons were shown to be associated with MIS in comparison to acute care surgeons at the multivariate analysis; as previously mentioned, this is related to the greater preparation that these types of surgeons often have in the MIS approach compared to emergency surgeons, and underlines again the need of having a solid preparation in laparoscopic skills, to be adopted also in emergency surgery.

Patients subjected to MIS were more likely to receive surgery with the formation of a primary anastomosis, compared to open surgery patients which were more commonly subjected to Hartmann’s procedure. This may be related to the worse clinical status of the patient that tends to undergo open surgery, as previously stated, which may not permit the creation of an anastomosis in safety. This result might be a bias to why patient who underwent laparoscopy had a better outcome in terms of morbidity and mortality, as the current literature suggests that primary anastomosis is superior to Hartmann’s procedure [19]. Di Saverio et al. proved in a study from 2016 that laparoscopy and intracorporeal anastomosis were possible in the emergency setting and could offer better results than open surgery, which was confirmed by the data of our study [20].

Our study also has some limits, such as the fact that it was built in a retrospective manner. This potential bias can however be balanced by the high number of patients that were enrolled in each center. Another limit could be the heterogeneity among the six selected centers since there was no pre-defined study protocol. However the aim of the study was to describe the real-life data from six high volume centers.

## Conclusion

In conclusion MIS is adopted in a small proportion of patients with left colon perforation. The adoption of minimally invasive techniques is limited by the severity of patients’ conditions, logistic factors, such as the time of surgery, and the skills of the operating surgeon which still have an important weight on the choice of surgical approach. MIS surgical approach was related to better outcomes with lower mortality and morbidity. Our data suggest the need for further studies investigating the role of organizational model and surgical training in management of emergency surgery.
